# Recurrence of Ectopic Cushing’s Syndrome 10 Years After Bilateral Adrenalectomy

**DOI:** 10.7759/cureus.11704

**Published:** 2020-11-25

**Authors:** Yusef Hazimeh, Zaynab Khalaf, Sablaa Ali, David Rayne

**Affiliations:** 1 Endocrinology, Lebanese University Faculty of Medicine, Beirut, LBN; 2 Radiology, Arnot Ogden Medical Center, New York, USA; 3 Endocrinology, Arnot Ogden Medical Center, New York, USA

**Keywords:** ectopic cushing syndrome, lung tumor, carcinoid, adrenalectomy

## Abstract

Ectopic Cushing’s syndrome is a severe form of Cushing disease. Treatment usually involves the resection of the adrenocorticotropic hormone producing tumor. In certain cases, bilateral adrenalectomy is carried out as a final resort in treatment. We present a patient who had a lung carcinoid tumor, which was producing adrenocorticotropic hormone and causing ectopic Cushing’s syndrome. Lung wedge resection failed to normalize cortisol level, and he had bilateral adrenalectomy. Ten years later, he had a recurrence of Cushing’s disease due to lymph node metastasis of his carcinoid tumor.

## Introduction

An elevated cortisol level, or hypercortisolism, can be due to several conditions that secrete cortisol or adrenocorticotropic hormone (ACTH) in large amounts. Patients with Cushing syndrome (CS) have typical features of hypercortisolism including abdominal striae, round face, fatigue, central obesity, hypertension, myopathy, osteoporosis, lipodystrophy, acne, hirsutism, oligomenorrhea, amenorrhea, and cognitive dysfunction including depression and memory loss [1].

Several etiological forms of CS can be distinguished: the ACTH-independent CS resulting from over-secretion of cortisol in the adrenal gland known as primary CS, the ACTH-dependent CS resulting from overproduction of ACTH from pituitary gland known as Cushing disease, and finally, the ACTH-dependent CS resulting from the overproduction of ACTH outside the pituitary gland, known as Ectopic Cushing Syndrome (ECS) [1-3]. Iatrogenic hypercortisolism is the most common cause of CS, followed by the ACTH-dependent Cushing disease.

Ectopic ACTH hyper-secretion from non-pituitary tumors was reported for the first time in 1928. It accounts for 10-20% of all cases of CS [2]. The majority of ectopic ACTH secretion originates from the lung, where 50% of cases were associated to small cell lung cancer and carcinoid tumor of the lung. The next most common origins include the thymus and the pancreas. This variety of organs renders the localization and treatment of this condition difficult and challenging [1-3].

The first step in diagnosing CS is to confirm the high cortisol level, which is mainly undertaken using three tests: 24-hour urinary cortisol level, midnight salivary cortisol level, and low-dose dexamethasone suppression test. The high-dose dexamethasone suppression test is mainly used to differentiate between the pituitary ACTH-secreting CS and ECS. MRI of the pituitary gland is the first radiological test to be carried out, in order to differentiate between these two entities causing ACTH-dependent cortisol hypersecretion. If MRI is negative, inferior petrosal sinus sampling (IPSS) is recommended by current guidelines [3].

We present herein, a rare challenging case of a 38-year-old male patient presenting with recurrent CS after bilateral adrenalectomy, which was found to be the result of ECS, secondary to carcinoid tumor of the lung.

## Case presentation

A 38-year-old male was diagnosed with Cushing's syndrome in 2003. He was found to have ectopic ACTH production, secondary to a right lung tumor. He underwent right middle lobe wedge resection in 2004. Pathology revealed a carcinoid tumor; symptoms persisted. Octreotide scan in 2005 showed recurrent lesion in the hilar region. He had a redo right thoracotomy that failed to locate any tumor. He underwent a bilateral adrenalectomy in 2006 and was put on maintenance-dose hydrocortisone. His symptoms were finally under control. He failed to follow up with the endocrinologist.

He was submitted to our practice in 2016, and was noted to have easy bruising, flushing, weight gain, and central obesity. His hydrocortisone was titrated down without relief. Workup for recurrence of his Cushing's revealed a 24-hour urine cortisol of 900 mcg (normal 3.5-45 mcg). ACTH level was 187 pg/ml (normal 10-60 pg/ml). Morning cortisol level post 1 mg dexamethasone suppression was 14.2 mcg/dl (normal <1.8 mcg/dl).

**Figure 1:**
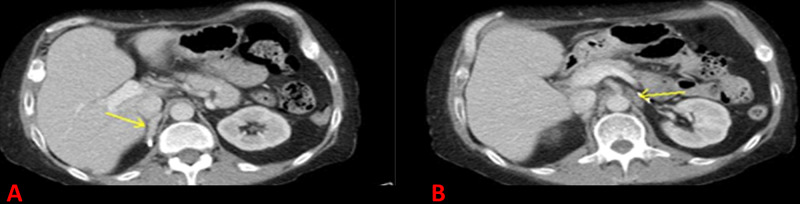
Computed tomography scan of the abdomen: (A) right adrenal gland (arrow), and surgical clip from previous adrenalectomy is seen posterior to the gland. (B) left adrenal gland (arrow).

Magnetic resonance imaging of the pituitary did not reveal any tumor. Computed tomography (CT) scan with adrenal protocol showed bilateral adrenal hyperplasia (Figure 1), which was deemed to be difficult to resect.

**Figure 2:**
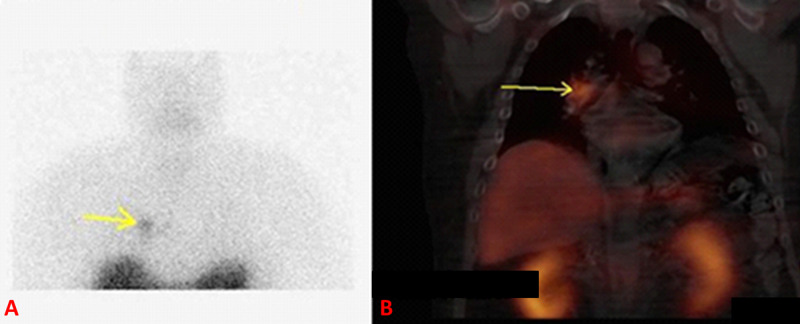
(A) Planar imaging of octreotide scan showing the right hilar carcinoid tumor (arrow), (B) single photon emission computed tomography of same lesion (arrow).

An octreotide scan with Single Photon Emission Computed Tomography (SPECT) was consistent with right hilar carcinoid tumor (Figure 2).

CT chest showed a small right hilar soft tissue density around the bifurcation of the right mainstem bronchus, in line with octreotide scan findings.

Ultrasound-guided endobronchial biopsy was consistent with a lymph node harboring a carcinoid tumor. This was found to be unresectable due to its small size. He was treated with octreotide and radiation therapy to right hilum. Treatment was successful, and the patient went into adrenal insufficiency, which was treated with hydrocortisone.

## Discussion

CS is associated with high morbidity and mortality [4]. The majority of cases are due to excessive ACTH production, named ACTH-dependent Cushing disease. While most of these cases are caused by a pituitary adenoma, ECS is caused by secretion of ACTH from non-pituitary tumor. ECS accounts for about 15-20% of patients with ACTH-dependent CS [5]. It is most often derived from small-cell carcinoma of the lung, or pulmonary carcinoid tumor [6].

Treatment of ECS is aimed at lowering ACTH level and normalizing cortisol level [7]. This is usually achieved via excision, irradiation, or medical therapy of carcinoid tumor [7]. In patients who fail these therapies, bilateral adrenalectomy is considered as a definitive treatment [8]. A 20-year follow-up survey of 43 patients with pituitary-dependent Cushing, who underwent bilateral adrenalectomy, showed satisfactory results and an advantage over medical therapy [9]. However, there is not enough long-term follow-up data on patients who underwent bilateral adrenalectomy due to ECS. Neuroendocrine tumors (NETs) of the lung (bronchial carcinoid) constitute 1-2% of primary lung neoplasm [10]. Although atypical NET shows an aggressive behavior, typical bronchial carcinoid shows a more indolent nature. They grow slowly but can metastasize to regional nodes in 4-20% of patients [11].

Surgery is the treatment of choice for resectable bronchial carcinoid tumors with good prognosis and survival [12]. Certain selected patients with unresectable tumors may benefit from adjuvant radiation therapy, chemotherapy, or somatostatin analogs [13]. Our patient underwent surgical excision of the carcinoid tumor of the lung in 2004. However, his ACTH was still elevated. He had a positive finding on octreotide scan two years later. This was likely a small lymph node that was not identified in the second surgery. He had bilateral adrenalectomy for a definitive “cure” for his ECS. He failed to follow up for eight years. His bronchial carcinoid was left untreated and therefore, the source of ACTH production was still intact. Excessive ACTH caused stimulation of remnant microscopic adrenal tissue. With time, new bilateral adrenal tissue (Figure 1) grew again, and the patient had recurrence of his ECS. He was not a candidate for a repeated bilateral adrenalectomy, or for surgical resection for bronchial lymph node metastasis. The patient benefitted from radiation therapy and octreotide.

## Conclusions

Patients who undergo bilateral adrenalectomy as a definitive treatment for ECS, should have long-term follow-up. Bronchial carcinoid tumors should be monitored and managed accordingly after adrenalectomy. Regrowth of remnant adrenal tissue is possible in the presence of long-term ACTH stimulation, and may lead to overt CS again. Treatment of ACTH-producing tumor with radiation therapy, along with octreotide, might be a good option for recurrent disease.
